# Draft genome sequences of three filamentous cyanobacteria isolated from brackish habitats

**DOI:** 10.7150/jgen.53678

**Published:** 2021-02-17

**Authors:** Joanne Sarah Boden, Michele Grego, Henk Bolhuis, Patricia Sánchez-Baracaldo

**Affiliations:** 1 School of Geographical Sciences, Faculty of Science, University of Bristol, Bristol, BS8 1SS, United Kingdom.; 2 Department of Marine Microbiology and Biogeochemistry, Royal Netherlands Institute for Sea Research, and Utrecht University, Den Hoorn, the Netherlands.

**Keywords:** *Spirulina sp. CCY15215*, * Halomicronema* sp. CCY15110, *Leptolyngbya* sp. CCY15150, cyanobacteria, genome, brackish

## Abstract

Brackish cyanobacterial genome sequences are relatively rare. Here, we report the 5.5 Mbp, 5.8 Mbp and 6.1 Mbp draft genomes of *Spirulina* sp. CCY15215, *Leptolyngbya* sp. CCY15150 and *Halomicronema* sp. CCY15110 isolated from coastal microbial mats on the North Sea beach of the island of Schiermonnikoog in the Netherlands. Large scale phylogenomic analyses reveal that *Spirulina* sp. CCY15215 is a large cell diameter cyanobacterium, whereas *Leptolyngbya* sp. CCY15150 and *Halomicronema* sp. CCY15110 are the first reported brackish genomes belonging to the LPP clade consisting primarily of *Leptolyngbya*, *Plectonema* and *Phormidium* spp. Further genome mining divulges that all new draft genomes contain, *ggpS* and *ggpP***,** the genes responsible for synthesising glucosylglycerol (GG), a compatible solute found in moderately salt-tolerant cyanobacteria.

## Introduction

Cyanobacteria (commonly known as 'blue-green algae') are oxygenic photoautotrophs which evolved in the Archaean (4 to ~2.5 billion years ago) 1, 2 and have since diversified into a wide range of habitats, including lakes, oceans, soil, rocks, geothermal springs and glaciers 3. Brackish species have been found living in estuarine environments 4, 5, but few genomes of brackish species have been sequenced 6. Some of these include *Nodularia spumigena* CCY9414 and *Aphanizomenon flos-aquae* from the Baltic Sea 7, 8 and four strains of *Microcystis aeruginosa* from Japan and the Netherlands 9-11.

Phylogenetic and trait evolution studies suggest there have been several transitions between saltwater and freshwater environments during the evolution of cyanobacteria 12. Intermediate stages of such transitions could have lived in brackish environments before acquiring the genetic mechanisms (e.g. synthesis of compatible solutes 13) necessary to tolerate higher salinity of the ocean or lower salinity of freshwater lakes and rivers inland. To understand these stages in more detail, we sequenced draft genomes of three new brackish and filamentous strains isolated from sediments on the island of Schiermonnikoog in The Netherlands: *Spirulina* sp. CCY15215, *Halomicronema* sp. CCY15110 and *Leptolyngbya* sp. CCY15150.

## Materials and Methods

Three strains of cyanobacteria were isolated from a microbial mat on the island of Schiermonnikoog in The Netherlands 14. Mono-phototrophic cultures were isolated by plating and grown in BA+ medium (https://www.ccy.nioz.nl/cyanobacteria_media) at 20°C under a 16 h: 8 h light:dark cycle with 10-20 μmol m^-2^ s^-1^ of white light.

Genomic DNA was extracted from 1.8 ml of each microbial culture using DNeasy ultraclean microbial kits (Qiagen, Germany) according to the manufacturer's instructions. Purified genomic DNA was stored in 10 mM Tris buffer at pH 8 and -80°C until further use.

Genomic DNA libraries for each strain were prepared using the TruSeq Nano LT kit (Illumina, Inc, Cambridge, UK). Whole genome sequencing was performed by the University of Bristol Genomics Facility, UK. Paired-end reads of 2x150 bps were obtained using a NextSeq® 500/550 (Illumina, San Diego, CA) with 300 cycles of the Mid Output Kit v2. Raw reads were quality trimmed using Trimmomatic v0.39 15 and de novo assembly performed with SPAdes v3.14.0 16. Short contigs containing less than 200 nucleotides were removed from the resulting assemblies. Cyanobacterial contigs were separated from other contigs using BLAST analysis and a de Bruijn graph visualisation approach that has previously been used to assemble draft genomes of *Phormidesmis priestleyi* BC1401 17 and *Leptolyngbya* sp. BC1307 18; see Supplementary Information Figures S1-S3. Genome completeness was measured based on comparisons with single-copy orthologs from cyanobacteria using BUSCO v3.0.2 19. All draft genomes were submitted to JGI IMG/ER 20 for annotation (GOLD Analysis Project IDs: Ga0438272, Ga0438271 and Ga0437567). Draft genome sequences have been deposited in DDBJ/ENA/Genbank repositories under accession numbers JACSWA000000000 for *Spirulina* sp. CCY15215, JACSWB000000000 for *Leptolyngbya* sp. CCY15150 and JACSWC000000000 for *Halomicronema* sp. CCY15110. These are stored within BioProject PRJNA658956 with BioSample accessions from SAMN15893520 to SAMN15893522.

The evolutionary relationships of our brackish strains with other cyanobacteria were estimated by performing maximum likelihood phylogenetic analysis of 139 orthologous proteins, SSU rRNA and LSU rRNA from 168 strains representing the entire diversity of the Phylum, Cyanobacteria. Similar methods have been implemented previously (see these studies for a more detailed description 6, 12, 21). Briefly, genomes were obtained from the NCBI RefSeq database (https://www.ncbi.nlm.nih.gov/refseq/). Protein and rRNA sequences were identified using the basic local alignment search tool (BLAST) for proteins or nucleotides respectively with an e value cut off <1×10^-25^ to remove anomalous hits. Each protein and rRNA were aligned using MAFFT v7.427 22 and gaps removed if present in 85% or more sequences. The phylogeny itself was constructed in IQ-TREE v1.6.7 23 using partitioned analyses which account for heterotachy and allow each protein and rRNA to evolve under a substitution model appropriate to that unique protein or RNA. Each of the substitution models were selected using Bayesian Information Criterion scores predicted by ModelFinder 24. Support for branching relationships were measured by calculating ultrafast bootstrap approximations with 1000 replicates 25. The resulting phylogeny was rooted with four representative strains of Vampirovibrionia, the sister phylum of Cyanobacteria 26.

## Results and Discussion

**Figure 1:**
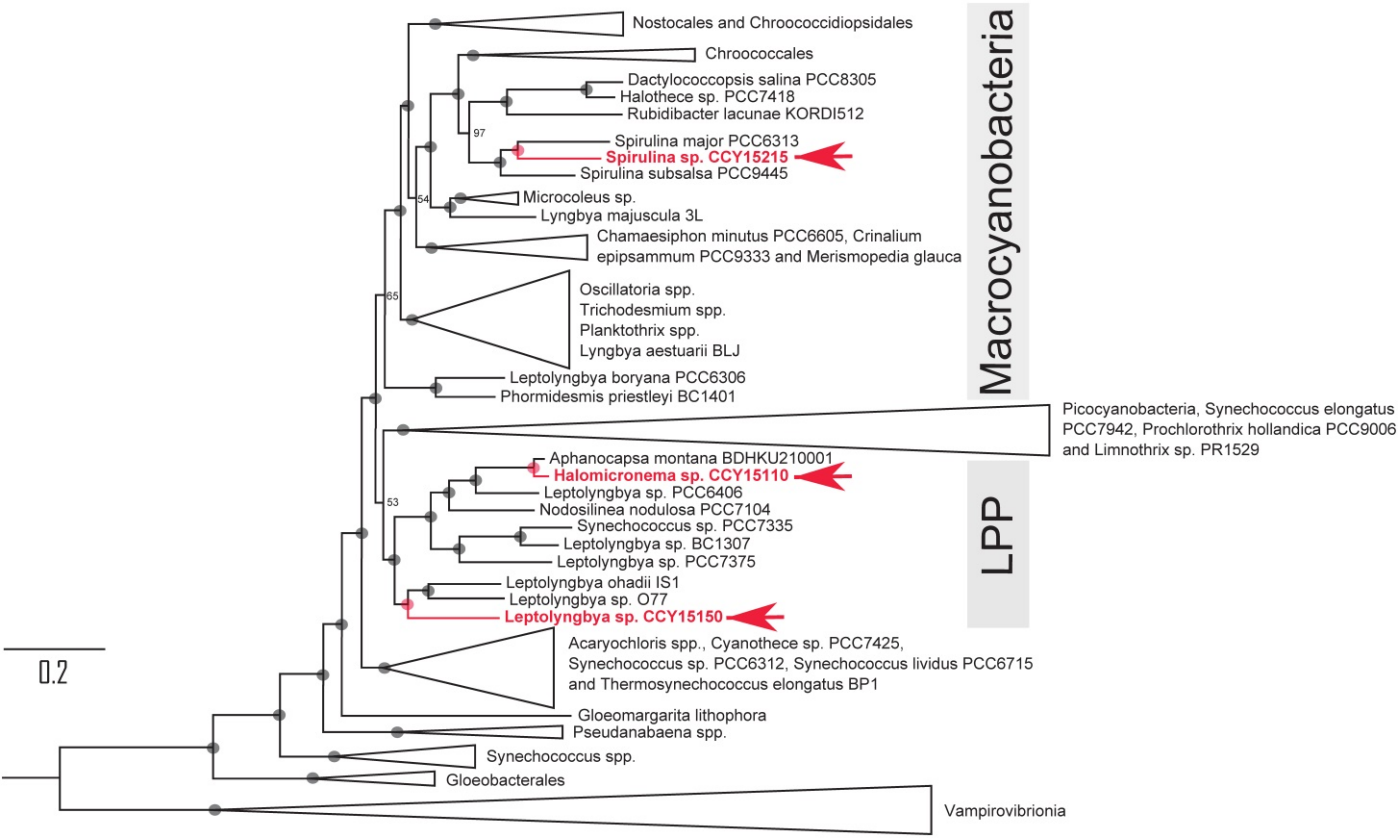
Phylogenetic tree showing the relationships of *Spirulina* sp. CCY15215, *Halomicronema* sp. CCY15110 and *Leptolyngbya* sp. CCY15150 (red) within the Phylum, Cyanobacteria. The tree was constructed from alignments of 139 orthologous proteins, SSU rRNA and LSU rRNA using Maximum Likelihood methodology implemented in IQ-TREE v1.6.7 23. It was rooted using four strains of Vampirovibrionia as the outgroup. Node labels represent ultrafast bootstrap approximations with circles representing values of 100. The scale bar represents an average of 0.2 substitutions per site. For a complete tree detailing all taxa that were included, see Supplementary Information Figure S4.

Each draft genome is composed from between 304 and 621 contigs with genome sizes ranging from 5.5 to 6.1 Mbps. Overall genome completeness is estimated at between 97.1 to 98.0% with N50 values in the range of 31,079 to 116,290 and GC content from 42.6 to 54.6% (Table 1). To find out how these strains are related to other cyanobacteria, phylogenomic analyses were conducted using 139 orthologous cyanobacterial proteins as well as SSU and LSU rRNA. They reveal that 2 of the 3 filamentous brackish strains are nested within the LPP clade previously defined by Sánchez-Baracaldo 6 (Figure 1, Supplementary Information Figure S4). The other, named *Spirulina* sp. CCY15215 is in a distant phylogenetic position within the Macrocyanobacterial clade defined by Sánchez-Baracaldo 12 (Figure 1). Macrocyanobacteria are ecologically diverse, but generally have large cells, ranging from 3 to 50 μm in diameter. Members of the clade vary from Nostocales to *Microcystis* spp. and *Oscillatoria* spp.

In particular, *Spirulina* sp. CCY15215 shares a recent common ancestor with two other *Spirulina* spp. that have slightly smaller genome sizes of 5.05 and 5.32 Mbp 27 compared to 5.5 Mbp for *Spirulina* sp. CCY15215. The closest relative, *Spirulina major* PCC6313 was also isolated from brackish waters 27. It was found in California, North America 27, more than 8000 km away from the Netherlands where *Spirulina* sp. CCY15215 was collected from. Their outgroup, *Spirulina subsalsa* PCC9445*,* has previously been cultured in artificial seawater 28. Since these close relatives of *Spirulina* sp. CCY15215 also grow in brackish and saltwater habitats, the most recent common ancestor of *Spirulina* spp. may also have been salt-tolerant.

Unlike *Spirulina* sp. CCY15215 which shares a common ancestor with brackish cyanobacteria, *Halomicronema* sp. CCY15110 is sister to a marine strain, named *Aphanocapsa montana* BDHKU 210001 (Figure 1). Phylogenetic analyses conducted using 16S SSU rRNA show that *Halomicronema* sp. CCY15110 is also closely-related to *Halomicronema excentricum str. Lakshadweep*, isolated from a marine habitat in India (Supplementary Information, Figure S5). The genus name 'Halomicronema' was therefore assigned to reflect this relationship.

To further investigate the mechanisms responsible for salt tolerance, protein BlastP searches were conducted for enzymes involved in the production of compatible solutes. Compatible solutes are molecules which aid osmoregulation in salty water 29. Their production is catalysed by a series of genome-encoded enzymes, including phosphatases (eg. GpgP, GgpP, Spp), synthases (eg. GpgS, GgpS, TreY, Sps), hydrolases (eg. TreZ) and methyltransferases (eg. GSMT and DMT). In order to make any single compatible solute, multiple enzymes are required. For example, an appropriate synthase and phosphatase must be present to combine the glucose group of one molecule with the carbohydrate group of another and then remove a phosphate ion to synthesise sucrose, glucosylglycerol (GG) or glucosylglycerate (GGA). Whereas, to synthesise trehalose and glycine betaine (GB), either a synthase and hydrolase or two different methyltransferases must be encoded respectively in the genome 29. To ensure we correctly identified the presence or absence of genes encoding these enzymes in each new genome, BlastP were conducted with query sequences from well-characterised cyanobacteria, including *Synechocystis* sp. PCC 6803,* Nostoc* sp. PCC 7120 and *Synechococcus* sp. PCC 7002 amongst others (Supplementary Information, Table S1).

Our genome mining reveals that all three brackish strains are genetically capable of making GG (Table 2). It has previously been hypothesised that GG-production is a characteristic of moderately halotolerant marine cyanobacteria 29. In addition to GG, *Leptolyngbya* sp. CCY15150 and *Halomicronema* sp. CCY15110 can make GGA (Table 2). Experimental studies measuring the accumulation of compatible solutes in cyanobacteria exposed to changing environmental conditions, suggest that GGA production increases in response to nitrogen limitation as well as increasing salinity 30, so its presence in *Leptolyngbya* sp. CCY15150 and *Halomicronema* sp. CCY15110 may help them to grow in lower nitrogen concentrations.

In addition to GG and GGA, *Leptolyngbya* sp. CCY15150 is genetically capable of making two further compatible solutes which may help it tolerate more saline conditions on the coastline. These are GB and sucrose (Table 2). Similarly large ranges of compatible solutes can be found in hypersaline strains 13, which is unusual given that *Leptolyngbya* sp. CCY15150 was isolated from a fresher, brackish environment 14. One explanation may lie in the changeability of intertidal habitats. Coastal microbial mats are subjected to alternate ebbs and flows of the tide and changing weather patterns (e.g. precipitation, humidity and temperature) which have the effect of changing salinity. For example, on hot days, after the tide recedes, water evaporates from the microbial mat, leaving the surrounding environment to become more and more saline until the next rainfall or influx of the tide. Metagenomic analyses show that during Summer, the hottest season of the year in Schiermonnikoog, *Leptolyngbya* spp. in general are more abundant than *Halomicronema* spp. 14. The presence of additional compatible solutes in *Leptolyngbya* sp. CCY15150 may go some way towards explaining this. Conversely, as draft genomes of *Halomicronema* sp. CCY15110 and *Spirulina* sp. CCY15215 both contain homologs of genes encoding one of two enzymes required for sucrose biosynthesis (SpsA but not Spp) and one of two enzymes required for GB biosynthesis (DMT but not GSMT, Table 2), there remains a small possibility that their complete genomes are sufficient to make additional compatible solutes.

Overall, draft genomes of three filamentous cyanobacteria expand the genomic representation of brackish strains in the tree of life and reveal that strains isolated from the same intertidal environment employ different strategies to acclimate to and tolerate variable salinities in a changing environment.

## Supplementary Material

Supplementary figures and tables.

## Figures and Tables

**Table 1:**
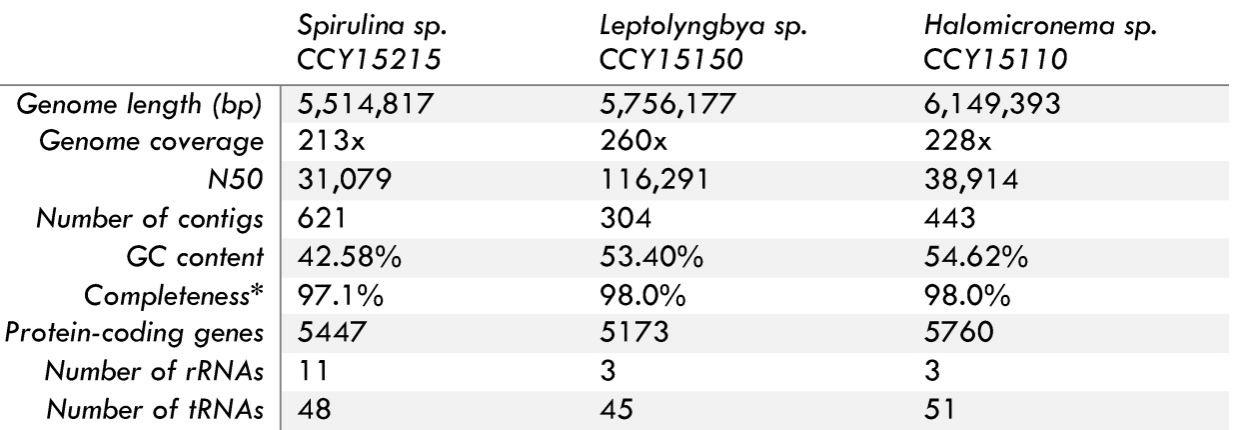
Genome Features

*Completeness was estimated using BUSCO v3.0.2 with lineage data from cyanobacteria 19.

**Table 2:**
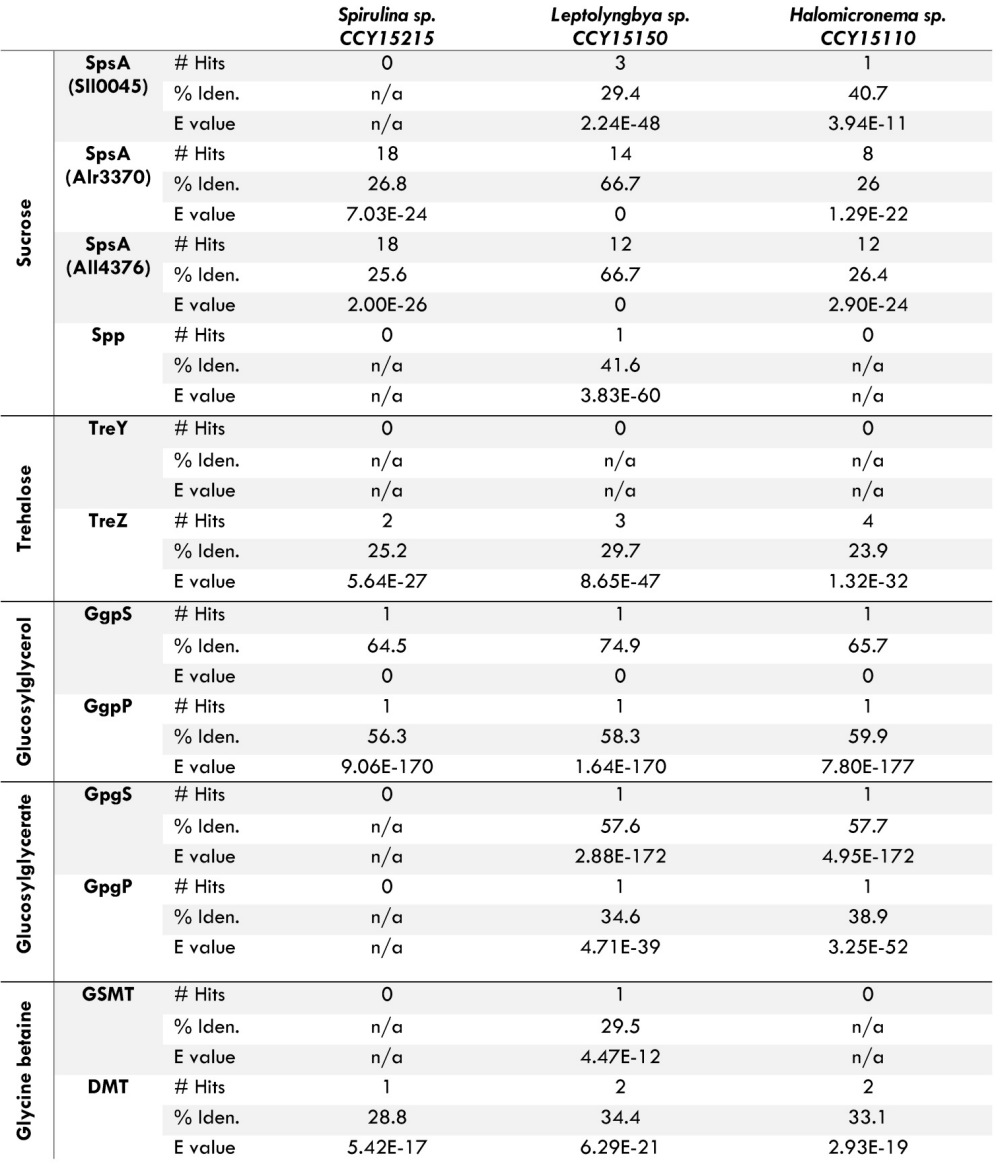
Results of BLASTP for genes encoding compatible solutes in three new draft genomes of cyanobacteria isolated from an overall brackish habitat

Homologs of genes required for the biosynthesis of sucrose (SpsA and Spp), trehalose (TreY and TreZ), glucosylglycerol (GgpS and GgpP), glucosylglycerate (GpgS and GpgP) and glycine betaine (GSMT and DMT) were found via BLASTP for genes identified by 29. All results have e values lower than 1x10^-10^. If multiple hits were found, statistics are reported for the hit with the smallest e value. % Iden. refers to percent identity.

## Abbreviations

BLAST: basic local alignment search tool
GG: glucosylglycerol
GGA: glucosylglycerate
GB: glycine betaine
SSU: small sub-unit
rRNA: ribosomal ribonucleic acid
LSU: large sub-unit
RNA: ribonucleic acid
