# Supplementary material for: Draft genome sequences of three filamentous cyanobacteria isolated from brackish habitats
Source: J Genomics. 2021 Feb 17;9:20–5. doi: 10.7150/jgen.53678 (PMC7893532; doi:10.7150/jgen.53678)
Supplement: Supplementary file 1 — Supplementary figures and tables. [file jgenv09p0020s1.pdf]

Supplementary Information: Draft Genome Sequences of Three Filamentous Cyanobacteria Isolated from Brackish Habitats

Joanne Sarah Boden[1], Michele Grego[2], Henk Bolhuis[2] Patricia Sánchez-Baracaldo[1]

[1] School of Geographical Sciences, Faculty of Science, University of Bristol, Bristol, BS8 1SS, United Kingdom

[2] Department of Marine Microbiology and Biogeochemistry, Royal Netherlands Institute for Sea Research, and Utrecht University, Den Hoorn, the Netherlands

Corresponding author(s)

Name(s): Joanne Sarah Boden and Patricia Sánchez-Baracaldo

E-mail(s): j.boden@bristol.ac.uk and p.sanchez-baracaldo@bristol.ac.uk

Includes:

Supplementary Figures S1 to S5

Supplementary Table S1

Figure S1: De Bruijn graph visualisation of assembled reads from the culture of *Halomicronema* sp. CCY15110. Nodes are coloured on a graduated scale according to sequencing depth of ≥83.36 (green) and ≤ 10.84 (red). Matches to known cyanobacterial genes are annotated with black text.

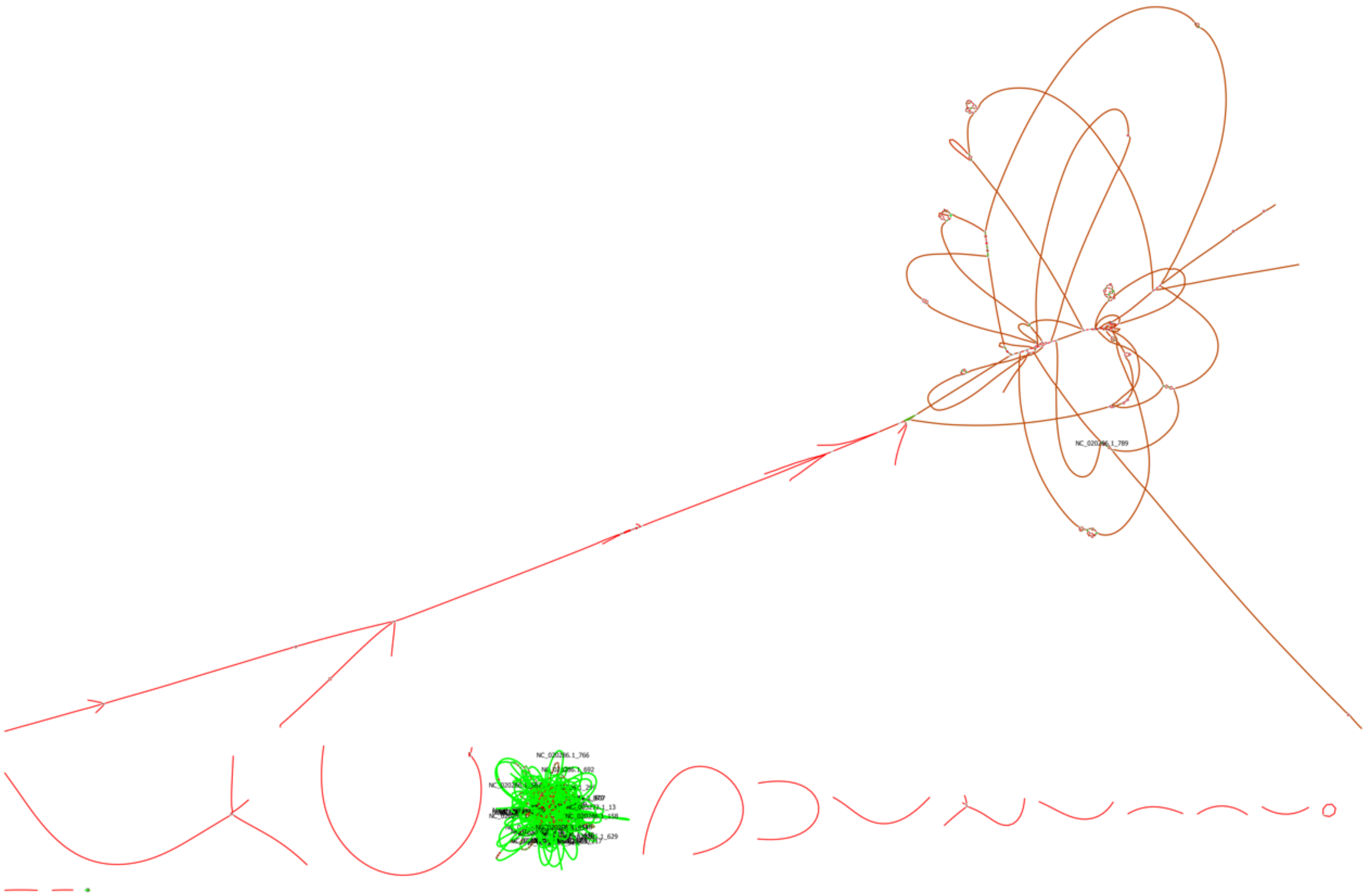

Figure S2: De Bruijn graph visualisation of assembled reads from the culture of *Leptolyngbya* sp. CCY15150. Nodes are coloured on a graduated scale according to sequencing depth of ≥65.3 (green) and ≤16.08 (red). Matches to known cyanobacterial genes are annotated with black text.

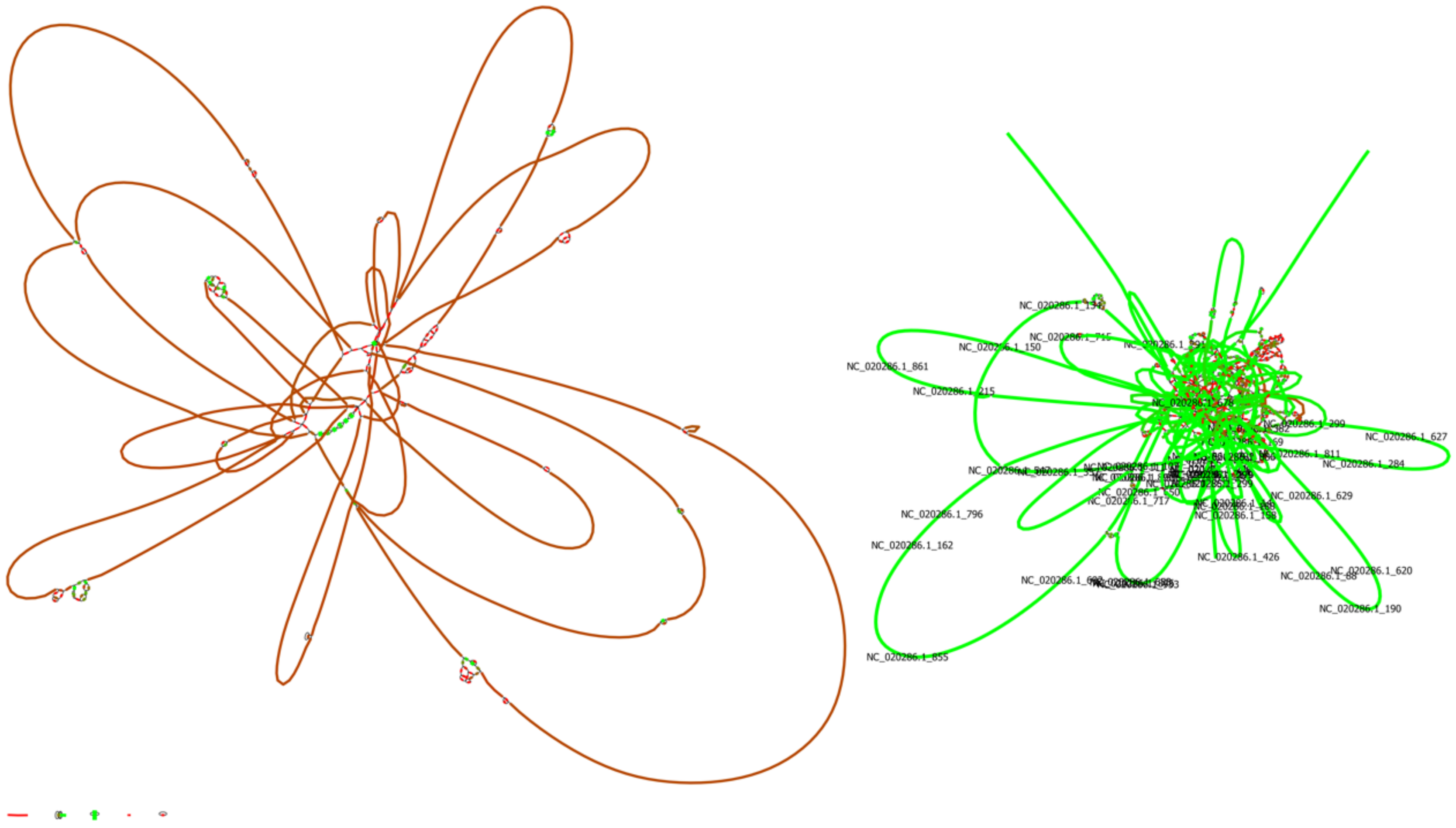

Figure S3: De Bruijn graph visualisation of assembled reads from the culture of *Spirulina* sp. CCY15215. Nodes are coloured on a graduated scale according to sequencing depth of ≥55.86 (green) and ≤16.93 (red). Matches to known cyanobacterial genes are annotated with black text.

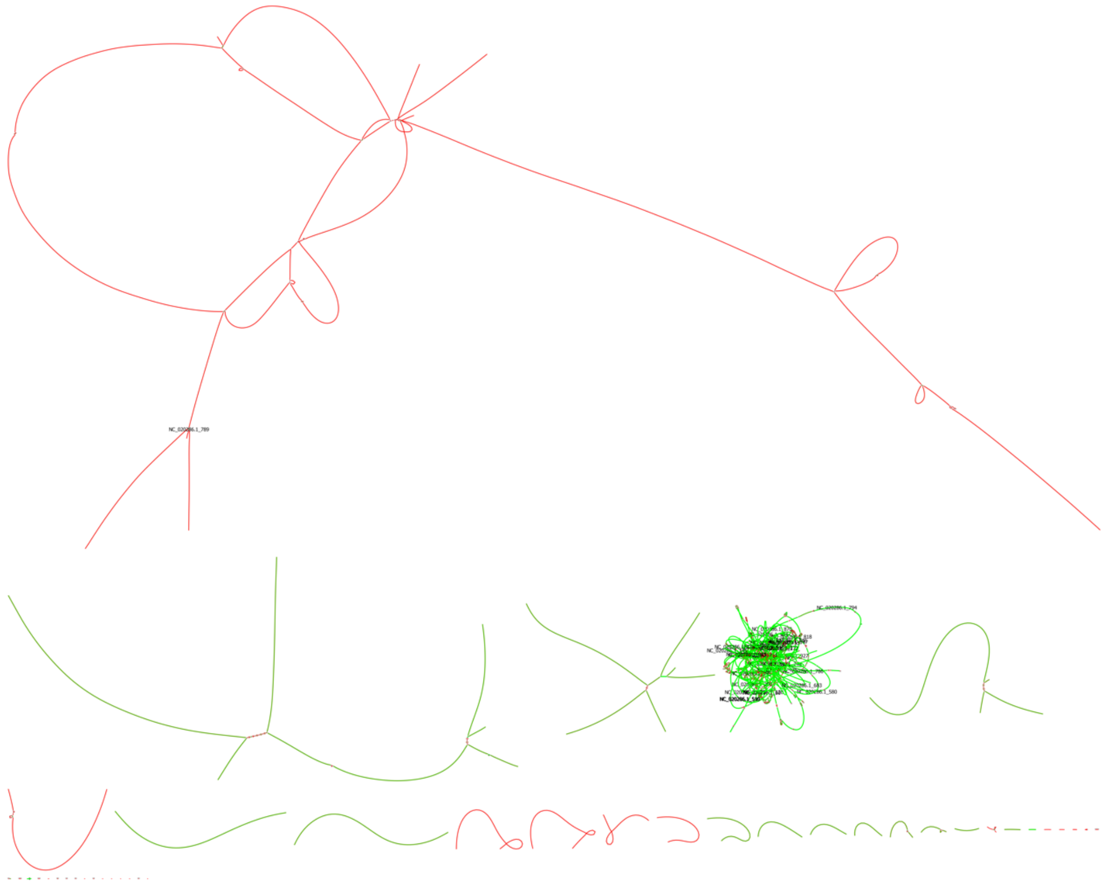

Figure S4: Maximum likelihood phylogeny of cyanobacteria, generated in IQ-TREE v1.6.7 (23) from an alignment of 139 proteins (53,242 amino acid positions), 16S rRNA (1,717 nucleotide positions) and LSU rRNA (3,117 nucleotide positions). Node labels represent UFBoot support values (25). Branch lengths represent the number of substitutions per site, with the scale bar representing an average of 0.2 substitutions per site.

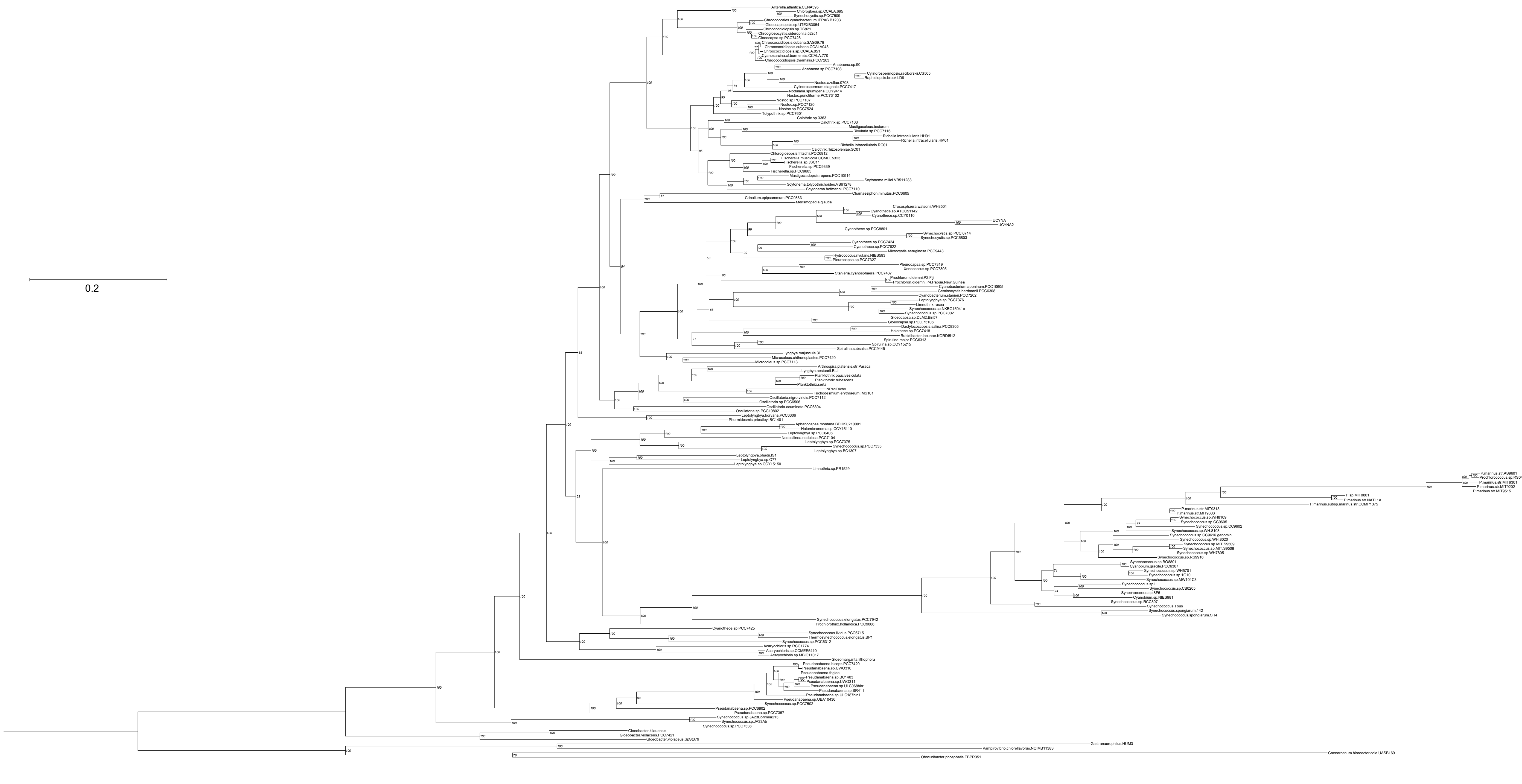

Figure S5: 16S SSU rRNA phylogeny of cyanobacteria, generated with maximum likelihood methodology implemented in IQ-TREE v1.6.1 (23) from an alignment of 1,717 nucleotide positions. Node labels represent UFBoot support values (25) less than 95, whereas node circles represent UFBoot support values higher than 95. Branch lengths represent the number of substitutions per site, with the scale bar representing an average of 0.2 substitutions per site. Draft genomes sequenced in this study are highlighted in red text with red arrows.

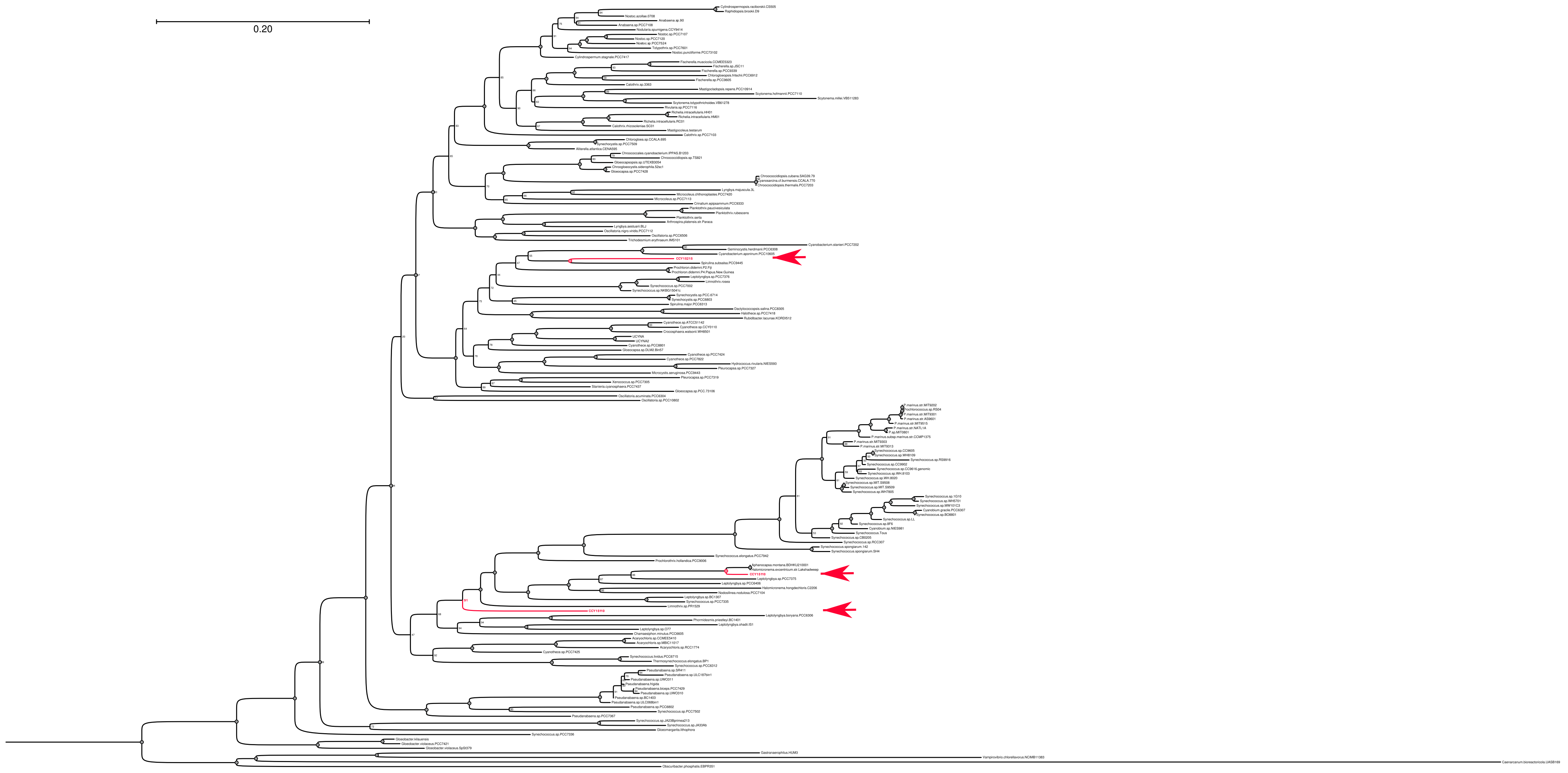

Table S1: Query sequences used for BlastP for compatible solutes

Compatible Solute	Enzyme	NCBI Accession Number	Source strain
Sucrose	SpsA (*Sll0045*)	AGF53073.1	*Synechocystis* sp. PCC 6803
	SpsA (*Alr3370*)	WP_044521731.1	*Nostoc* sp. PCC 7120
	SpsA (*All4376*)	WP_010998513.1	*Nostoc* sp. PCC 7120
	Spp	AGF52107.1	*Synechocystis* sp. PCC 6803
Trehalose	TreY	WP_010994344.1	*Nostoc* sp. PCC 7120
	TreZ	WP_044520508.1	*Nostoc* sp. PCC 7120
Glucosylglycerol	GgpS	AGF52040.1	*Synechocystis* sp. PCC 6803
	GgpP	AGF53289.1	*Synechocystis* sp. PCC 6803
Glucosylglycerate	GpgS	ACB00008.1	*Synechococcus* sp. PCC 7002
	GpgP	AAP99773.1	*Prochlorococcus marinus subsp. marinus* str. CCMP1375
Glycine betaine	GSMT	WP_015227493.1	*Halothece* sp. PCC 7418
	DMT	WP_015227494.1	*Halothece* sp. PCC 7418
